# Response Inhibition Deficits in Insomnia Disorder: An Event-Related Potential Study With the Stop-Signal Task

**DOI:** 10.3389/fneur.2018.00610

**Published:** 2018-08-07

**Authors:** Wenrui Zhao, Dong Gao, Faguo Yue, Yanting Wang, Dandan Mao, Xinyuan Chen, Xu Lei

**Affiliations:** ^1^Sleep and Neuroimaging Center, Faculty of Psychology, Southwest University, Chongqing, China; ^2^Key Laboratory of Cognition and Personality of Ministry of Education, Chongqing, China; ^3^Sleep Psychology Center, Daping Hospital, Army Medical University, Chongqing, China

**Keywords:** insomnia disorder, stop-signal task, response inhibition, event-related potentials, P3

## Abstract

**Background:** Response inhibition is a hallmark of executive function, which was detected impaired in various psychiatric disorders. However, whether insomnia disorder (ID) impairs response inhibition has caused great controversy.

**Methods:** Using the auditory stop-signal paradigm coupled with event-related potentials (ERPs), we carried out this study to examine whether individuals with ID presented response inhibition deficits and further investigated the neural mechanism correlated to these deficits. Twelve individuals with ID and 13 matched good sleepers (GSs) had participated in this study, and then they performed an auditory stop-signal task (SST) in the laboratory setting with high density EEG recordings.

**Results:** The behavioral results revealed that compared to GSs, patients with ID presented significantly longer stop-signal reaction time (SSRT), suggesting the impairment of motor inhibition among insomniacs. Their reaction time in go trials, however, showed no significant between-group difference. Considering the electrophysiological correlate underlying the longer SSRT, we found reduced P3 amplitude in patients with insomnia in the successful stop trials, which might reflect their poor efficiency of response inhibition. Finally, when we performed exploratory analyses in the failed stop and go trials, patients with ID presented reduced Pe and N1 amplitude in the failed sop trials and go trials respectively.

**Discussion:** Taken together, these findings indicate that individuals with ID would present response inhibition deficits. Moreover, the electrophysiological correlate underlying these deficits mainly revolves around the successful stop P3 component. The present study is the first to investigate the electrophysiological correlate underlying the impaired response inhibition among insomniacs.

## Introduction

Insomnia disorder (ID) has been one of the most prevalent and common psychophysiological disorders. According to International Classification of Sleep Disorders 3rd edition (ICSD-3), ID is defined as a persistent difficulty with sleep initiation, duration, consolidation, or quality that occurs despite adequate opportunity and circumstances for sleep, and results in some form of daytime impairment (1). They often complain about impaired cognitive function such as emotional processing (2, 3), memory consolidation (4, 5), vigilance (6, 7), selective attention (8–11), while inconsistent results were still found in executive function. A literature review of executive function found that the majority of studies failed to find its impairment in primary insomnia (12). But another meta-analysis study pointed out that individuals with insomnia exhibited impairments only in some complex tasks (for example, working memory, and cognitive flexibility), which were generally related to the integrity of the prefrontal cortex (13). However, evidence from functional neuroimaging seemed to favor the viewpoint of impaired executive function. Increasing studies had a tendency to recognize the existence of abnormal brain morphometry and reduced brain metabolism in prefrontal cortex among patients with insomnia, which manifested the probable deficit of executive function associated with this prefrontal dysfunction (14–16).

Response inhibition is a hallmark of executive function, and one of the representative psychological tasks to assess it is the stop-signal task (SST). SST is a two-choice reaction task in which participants are typically instructed to respond to a primary go stimulus as fast as possible, unless a stop-signal is occasionally and unexpectedly presented to instruct the participants to suppress the go response (17, 18). This paradigm, buttressed by the horse-race model, is an effective tool to investigate response inhibition in the laboratory setting (19). In the horse-race model, stop-signal performance depends on a race between a “go process” (triggered by a go stimulus) and a “stop process” (triggered by a stop-signal). Response inhibition is determined by the finishing time of two processes: if the go process ends before the stop process, the response is executed; contrarily, if the stop process finishes before the go process, the response is inhibited (20, 21). SST allows the estimation of response execution time to go stimuli and the latency of stop process (known as the stop-signal reaction time, SSRT). The latter is proved to reflect the speed of response inhibition.

There are numerous researchers who used the SST to assess the response inhibition in various psychiatric disorders, such as attention-deficit hyperactivity disorder (22, 23), obsessive-compulsive disorder (24, 25), schizophrenia (26, 27), and eating disorder (28). However, to our knowledge, only two studies had ever adopted the stop-signal paradigm to investigate the response inhibition in patients with ID. One of them employed the auditory SST to investigate the response inhibition process in primary insomnia and obstructive sleep apnea syndrome (OSAS). This study claimed that compared to controls, only OSAS presented impaired response inhibition (i.e., longer SSRT), but there was no significant SSRT difference between insomniacs and normal sleepers (29). Another study, however, reported a completely different result. Covassin and his colleagues found response inhibition deficits in insomnia patients (i.e., longer SSRT), accompanied by high levels of cardiovascular arousal (30). The discrepancy between the above two studies may partially result from clinical heterogeneity and the different methods in computing SSRT.

Previous functional neuroimaging studies had explored the neural substrates of response inhibition. In the SST, the go process typically activated a cortico-basal-ganglia-thalamocortical circuit, which included frontal, striatal, pallidal, and motor cortical regions; while the stop process mainly activated a fronto-basal-ganglia circuit, which included inferior frontal gyrus (IFG), middle frontal gyrus, medial frontal gyrus, subthalamic nucleus and striatum (17, 31, 32). Among these regions, prefrontal cortex, especially the right IFG, was considered to be the crucial region that contributed to response inhibition (33–35). Prefrontal hypoactivation and atrophy, for example, the reduced gray matter concentrations in bilateral IFG, had been substantiated by the majority of previous studies (15, 16, 36, 37), although contradictory results also were found in few studies due to the clinical heterogeneity or the technical diversity (38). Thus prefrontal dysfunction may be the neural correlate of the impaired response inhibition in insomniacs. Nevertheless, the poor temporal resolution of functional magnetic resonance imaging (fMRI) makes it difficult to determine the neural mechanism underlying insomniacs' poor response inhibition. Event-related potentials (ERPs), with its merit in temporal resolution, may be an optimal measure to provide insight into the spatiotemporal dynamics of response inhibition deficits in ID.

Several ERP components linked to response inhibitory processing: firstly the N1 component, a negative potential peaking around 100–150 ms with a central distribution; and then P3, a positive potential peaking around 300–400 ms with a fronto-central or central distribution (39, 40). The auditory N1 would be taken into consideration since it originates from the auditory cortex. This component indicated early orientation or selective attention to the stop-signal, which would affect subsequent inhibitory processing (39, 41). The P3 component was believed to have a specific association with the monitoring of the success of inhibitory processing (42, 43). Moreover, in the failed inhibition trials, we also focused on two error-related ERP components time-locked to the overt response. They were typically observed if subjects failed to stop the primary go response. The first component was known as error-related negativity (ERN), a sharp negative wave peaking around 60–80 ms after a button press, with the maximal amplitude in the frontal area. Another positive potential named error-positivity (Pe) would appear after the ERN showing up, which presented a posterior-central distribution. The ERN-Pe complex had been found to be functionally associated with error detection and adjustment of post-error decisions (44). Hitherto, to our knowledge, no study has investigated the above components underlying dysfunctional response inhibition in insomnia patients. In the present study, we placed a high value on N1 and P3 components time locked to stop-signal in the successful stop trials. Furthermore, exploratory analyses were also performed in both N1 and P3 components in the go trials, as well as the ERN-Pe complex in the failed stop trials, to investigate other possible factors resulting in response inhibition deficits in patients with ID.

Taken together, through using the auditory stop-signal paradigm and ERPs, the primary aim of our study was to address two questions: whether individuals with insomnia would present response inhibition deficits, and the electrophysiological correlate underlying these deficits. Based on previous studies, our hypotheses were as following: the impaired response inhibition in patients with ID, in comparison with that of the control group, might be reflected as a longer SSRT in behavior performance; the electrophysiological mechanism underlying the longer SSRT might correlate to the reduced N1 and P3 amplitude, and/or longer N1 and P3 latency in the successful stop trials.

## Materials and methods

### Participants

Thirteen good sleepers (GSs, 8 males; mean age = 41.3 years, S.D. = 12.4) and 12 patients with insomnia disorder (IDs, 7 males, mean age = 49.1 years, S.D. = 7.6) had participated in this experiment. All IDs were recruited from Department of Sleep Psychology Center, Daping Hospital, Army Medical University. GSs were recruited from the local community through advertisements. Some questionnaires including Pittsburgh Sleep Quality Index (PSQI) (45), Self-Rating Depression Scale (SDS) (46), Self-Rating Anxiety Scale (SAS) (47), and Barratt impulsiveness scale (BIS) (48), were completed to assess sleep quality and emotion state. Two experienced psychiatrists (author DG and FY) conducted the semi-structured clinical interviews to exclude any history of psychiatric and sleep disorders. All IDs met the following criteria: (1) age between 18 and 65; (2) conforming to the definition of ID by International Classification of Sleep Disorders-3; (3) PSQI score ≥7; (4) the duration of insomnia ≥1 year; (5) no inborn or other acquired psychiatric and physiological diseases, or any other sleep disorders (including hypersomnia, parasomnia, sleep-related breathing, sleep-related movement, or circadian rhythm sleep disorders); (6) free of any psychoactive medication for at least 2 weeks prior to and during the study participation. Besides, all GSs met the following criteria: (1) having good sleeping habits and no difficulty with sleep initiation and/or maintenance; (2) no history of swing shift, shift work, or sleep complaints; (3) no consumption of psychoactive drugs for at least 2 weeks prior to the study; (4) PSQI score < 7, the standardized score of SDS and SAS < 50. Moreover, participants gave their written informed consent after a detailed explanation of our study protocol and were then compensated for their participation. The study was approved by the Ethics Committee of the Southwest University and Army Medical University, and all procedures involved were executed in accordance with the sixth revision of the Declaration of Helsinki.

## Procedure

### Polysomnography

All participants were asked to arrive at the sleep laboratory in 18:00 p.m. to prepare for EEG recording, and then they were asked to sleep about 8 h from “lights out” (22:00–24:00) to “lights on” (06:00–08:00) with standard Polysomnography (PSG) recordings at Department of Sleep Psychology Center, Daping Hospital of Army Medical University. PSG parameters were used to exclude any other sleep disorders. Participants, who have periodic limb movements in sleep or a sleep apnea index per total sleep time (TST) of more than 5.0/h, were excluded in this study. Sleep was recorded by 16-channel Nihon Kohden EEG-polysomnograph, which included 6 EEG leads with bipolar re-referencing (F3-A2; F4-A1; C3-A2; C4-A1; O1-A2; O2-A1), electrooculography (horizontal and vertical), and submental electromyography. Sleep stages were scored visually by two experienced raters (authors YW and DM) according to American Academy of Sleep Medicine criteria (49). By monitoring abdominal and thoracic effort, nasal airflow, oximetry, and bilateral tibialis anterior electromyography, any participants detected with apnea and periodic limb movements would be excluded from this study. The sleep parameters were shown in Table 1.

**Table 1:** Descriptive statistic of demographic, clinical and polysomnographic data.

**N**	**Insomnia disorders**	**Good sleepers**	**T**	***p***
	**12 (7 males)**	**13 (8 males)**		
Age	49.1 ± 7.6	41.3 ± 12.4	1.869	*0.074*
BMI [kg/m^2^]	22.5 ± 3.0	21.8 ± 2.5	0.624	0.538
Education [y]	9.9 ± 2.7	12.8 ± 4.1	−2.078	*0.052*
Insomnia duration [M]	123.7 ± 57.6	–	–	–
PSQI	15.5 ± 2.8	3.9 ± 1.8	12.459	**< 0.001**
BIS	37.4 ± 14.4	30.2 ± 9.2	1.513	0.144
BIS-M	36.5 ± 16.5	30.2 ± 12.5	1.078	0.292
BIS-C	36.9 ± 15.6	30.0 ± 11.5	1.265	0.219
BIS-P	39.0 ± 22.3	30.4 ± 11.8	1.187	0.252
SDS	55.0 ± 13.7	34.2 ± 6.7	4.760	<**0.001**
SAS	51.3 ± 9.7	30.7 ± 3.7	6.896	<**0.001**
**POLYSOMNOGRAPHIC DATA**				
TST [min]	399.6 ± 65.6	391.6 ± 71.4	0.291	0.774
SEI [%]	75.6 ± 14.6	84.8 ± 11.2	−1.759	*0.092*
SL [min]	15.7 ± 9.3	11.6 ± 9.4	1.093	0.286
WASO [min]	112.3 ± 75.8	46.0 ± 40.0	2.701	**0.016**
REML [min]	156.0 ± 116.5	114.2 ± 63.0	1.103	0.286
S1 [%]	24.4 ± 13.7	18.6 ± 11.6	1.145	0.264
S2 [%]	54.6 ± 12.8	53.2 ± 12.9	0.264	0.794
SWS [%]	5.0 ± 4.4	9.6 ± 8.0	−1.800	*0.088*
REM [%]	16.0 ± 8.0	18.7 ± 5.3	−1.008	0.324

*Significant results are presented in bold (p < 0.05). Marginally significant results are evidenced in italics. BMI, Body Mass Index; BIS, Barratt Impulsiveness Scale; BIS-M, the dimension of Motor Impulsiveness of BIS; BIS-C, the dimension of Cognitive Impulsiveness of BIS; BIS-P, the dimension of No Planning Impulsiveness of BIS; PSQI, Pittsburgh Sleep Quality Index; SAS, Self-Rating Anxiety Scale; SDS, Self-Rating Depression Scale; TST, Total Sleep Time; SEI, Sleep Efficiency Index; SL, Sleep Latency; WASO, Wake After Sleep Onset; REML, Rapid Eye Movement Latency; S1, Sleep Stage 1; S2, Sleep Stage 2; SWS, Slow Wave Sleep; REM, Rapid Eye Movement*.

### Stop-signal task

**Figure 1:**
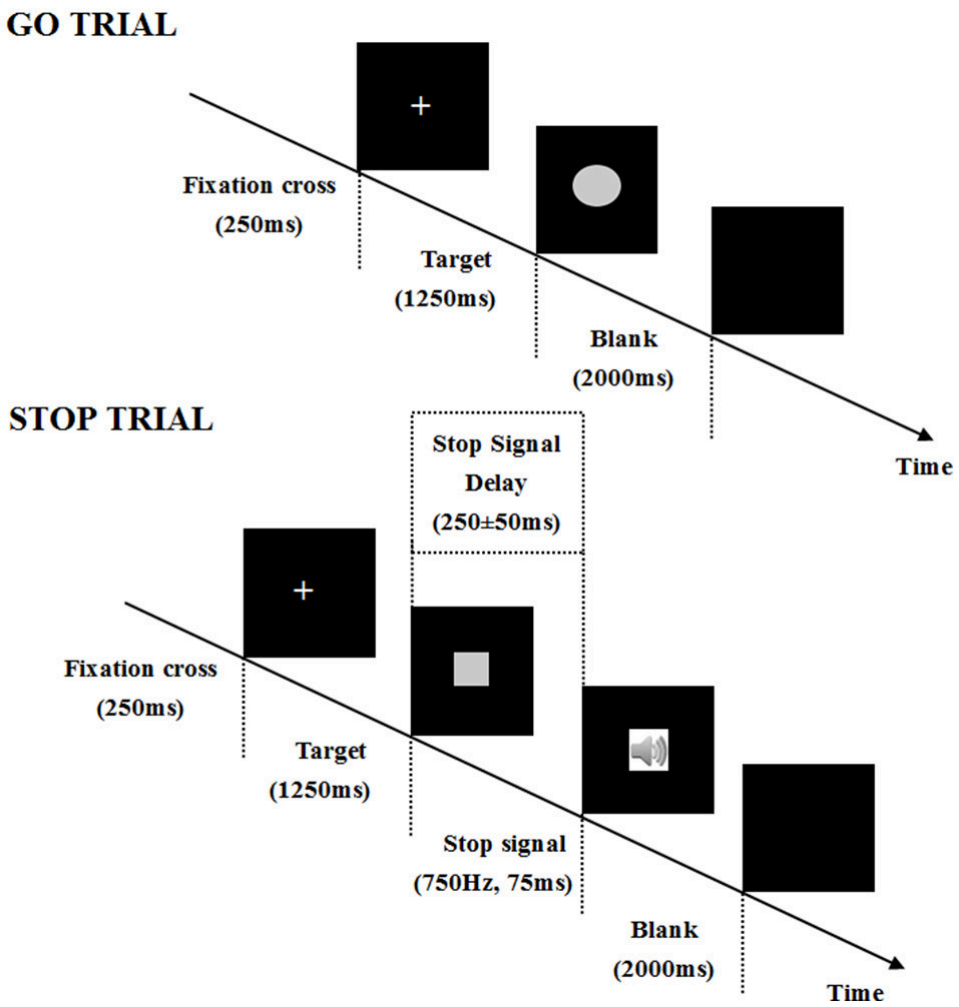
Depiction of a go and a stop trial course in the stop-signal task. In this paradigm, subjects are asked to perform an auditory two-choice reaction task. In the go trials, participants need discriminate the shape of a stimulus (a square corresponds to a left response “Q” and a circle corresponds to a right response “P”). Each trial begins with a central fixation cross (250 ms), then a square or circle is presented for 1,250 ms in a randomized order. The inter-trial interval is a blank screen of 2,000 ms. In the stop trials, occasionally, a tone (i.e., the stop-signal, 750 Hz, 75 ms) is presented shortly after the stimuli onset (a square or circle). Subjects are instructed to withhold their response when the tone occurs. In the stop-signal trials, the stop-signal is presented after a variable stop-signal delay (SSD; i.e., the delay between the onset of a go stimulus and the onset of a stop-signal). SSD is initially set at 250 ms and is adjusted dynamically with the staircase tracking procedure: when inhibition is successful, SSD increases by 50 ms; when the inhibition is unsuccessful, SSD decreases by 50 ms.

The auditory SST, programmed with reference to Verbruggen's study (18), was applied into our experiment (Figure 1). This experiment consisted of seven blocks, and every block comprised 64 trials (48 go trials and 16 stop trials). The first block was regarded as practice. All subjects were right-handed and comfortably seated in a well-lit, temperature-controlled, and soundly attenuated room. They were instructed to press a corresponding button on the keyboard as quickly and accurately as possible (go trials). When the tone occurred, they had to refrain from making any response at full strain (stop trials).

### EEG recording

Continuous scalp EEG was recorded by 63 Ag/AgCI active electrodes mounted within an elastic cap, based on the extended 10–20 international electrode placement system (Brain Products GmbH, Steingrabenstr, Germany), and two additional electrodes were used as reference and ground. The electrooculogram (EOG) was simultaneously recorded using two electrodes. One was placed below the left eye and another at the outer canthus of the right eye. The electrical signals were recorded at 500 Hz with an online 0.01–100 Hz band-pass filter, including a 50 Hz notch filter. The electrode impedance was kept below 5 kΩ after careful preparation.

### Statistical data analysis

The preprocessing was conducted with the assistance of custom-made MATLAB (R2014, The MathWorks, Inc.,) scripts supported by EEGLAB. All channels were firstly re-referenced offline to the average of the left and right mastoids. Then the EEG data were digitally filtered from 0.1 to 45 Hz via a conventional finite impulse response (FIR) filter. ERP epochs were defined from −100 to 900 ms (stimulus-locked around the stimulus in the go trials or the tone in the successful stop trials) and −500 to 900 ms (response-locked around the button press in the failed stop trials). In the go and successful stop trials, the epochs were baseline-corrected using a 100 ms pre-stimulus window, whereas in the failed stop trials, the epochs were aligned to a 500 ms pre-response window. Some epochs with ocular, muscular, and other types of artifacts were identified and then excluded from further analysis. This study also used the independent component analysis (ICA) to remove the remaining EOG artifacts according to participants' scalp maps and activity profile. As a result, on average, 274 trials for the go condition, 54 trials for the successful stop condition, and 41 trials for the failed stop condition were considered qualified for further analysis. There was no between-group difference in the number of the remaining trials. After careful inspection of the ERP waveforms and the scalp topography in every subject, we selected N1 (80–180 ms) and P3 (230–400 ms) locked to the stimulus presentation in the go trials and stop-signal in the successful stop trials. Moreover, we also focused on the components of ERN (0–100 ms) and Pe (150–300 ms) locked to the button press in the failed stop trials. The P2 and N2 components were not quantified since they were small in magnitude, which was common for auditory evoked ERPs (39, 50). Mean voltage amplitude and peak latency (i.e., the time interval between stimulus or response onset and maximal amplitude) in these component-specific windows were used for further statistical analysis. To increase the signal-to-noise ratio, six regions of interest (ROIs) were selected by averaging all neighbor electrodes: (1) AF3, F7, F3, Fz, AF4, F4, F8 (frontal), (2) FC5, FC1,C3,Cz, FC2, FC6, C4 (central), (3) CP5, CP1, P3, CP2, CP6, P4 (parietal), (4) AF3, F7,F3, FC5, FC1, C3, CP5, CP1, P3 (left), (5) Fz, Cz, Pz (midline), (6) AF4, F4, F8, FC2, FC6, C4, CP2, CP6, P4 (right). These electrodes and regions were selected based on the parameters defined in previous studies (23, 40, 51).

SPSS 16.0 was used as the statistical analysis toolbox. Two-sample *T*-test was used to find the between-group difference from demographic, clinical, and polysomnographic parameters. Two-way repeated measures ANOVAs were conducted to analyze amplitudes and latencies of these ERP components with ROIs as the within-subject factor, group (GS vs. ID) as the between-subject factor, and age and education level as covariates. The Greenhouse–Geisser correction was used to adjust the effects for any violation of sphericity. The study also involved Bonferroni correction bearing a corrected *p* < 0.0125 threshold into adjusting of multiple applications of ANOVA and Fisher's least significant difference (LSD) tests were used for *post hoc* comparisons. Additional bootstrap analyses were also carried out to assess the between-group differences of the behavioral data and the correlations between these behavioral parameters and P3 amplitude in the successful stop trials. All significant thresholds were set at *p* < 0.05 except the ANOVAs. The bootstrapping method is suitable to fix limitation caused by the small sample size and thus the statistical bias can be reduced. The bootstrap results in our study were based on 1,000 bootstrap samples with age and education as covariates. All data involved in this study are available upon request.

## Results

### Demographic data and sleep parameters

Descriptive statistics on demographic data and sleep parameters of the two groups were shown in Table 1. The two groups did not differ in BMI, BIS and the three subscales of BIS. Although the age and education levels presented marginally significant differences between two groups, they were used as covariates in all the following statistical analyses. As we expected, compared to GSs, patients with ID presented higher scores in PSQI, SAS, and SDS, which suggested poorer sleep quality and worse mood states in insomniacs.

Considering the PSG parameters, insomnia patients showed significantly increased time on wake after sleep onset (WASO). Moreover, marginally significant between-group differences were also observed in the sleep efficiency index (*p* = 0.092) and the percentage of slow wave sleep (*p* = 0.088). No significant difference was found in any other parameters.

### Behavioral results

**Figure 2:**
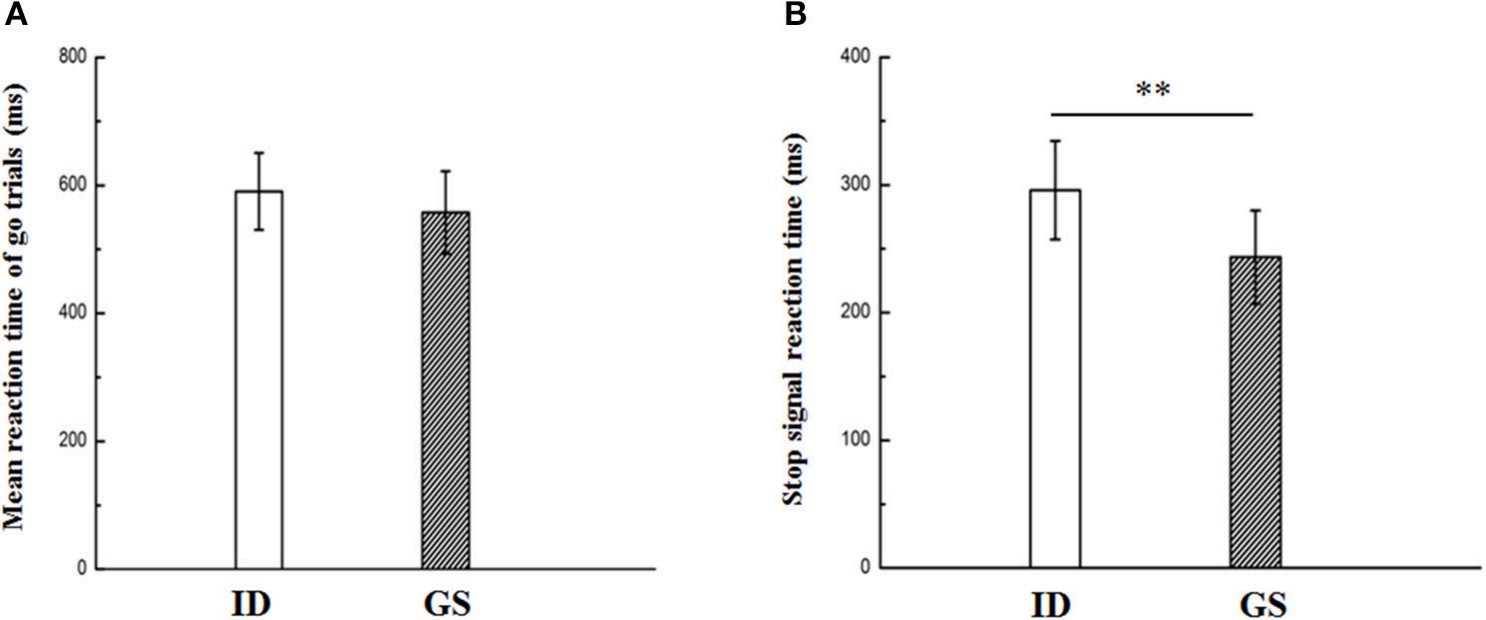
Reaction time (Go RT, **A**) in go trials and stop-signal reaction time (SSRT, **B**) in patients with insomnia disorder (ID) and good sleepers (GS). ** Indicates significant level of *p* < 0.05.

As illustrated in Figure 2B, significantly longer SSRT was found in IDs compared with that of GSs (*t*_(23)_ = 3.496, *p* = 0.002). The bootstrap result also revealed a stable and significant between-group difference on SSRT (*p* = 0.038 with 95% confidence intervals between 5.37 and 70.55). No significant between-group effect was found for go accuracy, stop accuracy, omissions, choice errors, false alarms, go RT, and SSD (Figure 2A and Table 2). These results suggested the impaired motor inhibition in patients with ID, but the motor response was relatively intact for them.

**Table 2:** Descriptive statistic of the stop-signal task.

	**Insomnia disorders**	**Good sleepers**	**T**	***p***
Go accuracy (%)	0.98 ± 0.01	0.99 ± 0.01	−0.634	0.532
Stop accuracy (%)	0.56 ± 0.07	0.57 ± 0.07	−0.151	0.881
Omissions (%)	0.01 ± 0.03	0.005 ± 0.006	1.332	0.196
Choice errors (%)	0.009 ± 0.01	0.008 ± 0.009	0.043	0.966
False alarms (%)	0.44 ± 0.07	0.43 ± 0.07	0.151	0.881
Go RT (ms)	590.5 ± 60.1	557.6 ± 64.6	1.315	0.201
SSRT (ms)	295.9 ± 38.5	243.4 ± 36.6	3.496	***0.002***
SSD (ms)	294.6 ± 63.5	311.5 ± 73.9	−0.609	0.549

*Significant result is presented in bold (p < 0.05). Go RT, Reaction Time of Go Trials; SSRT, Stop-signal Reaction Time; SSD, Stop-signal Delay*.

### Stop-signal ERPs in the successful stop trials

**Figure 3:**
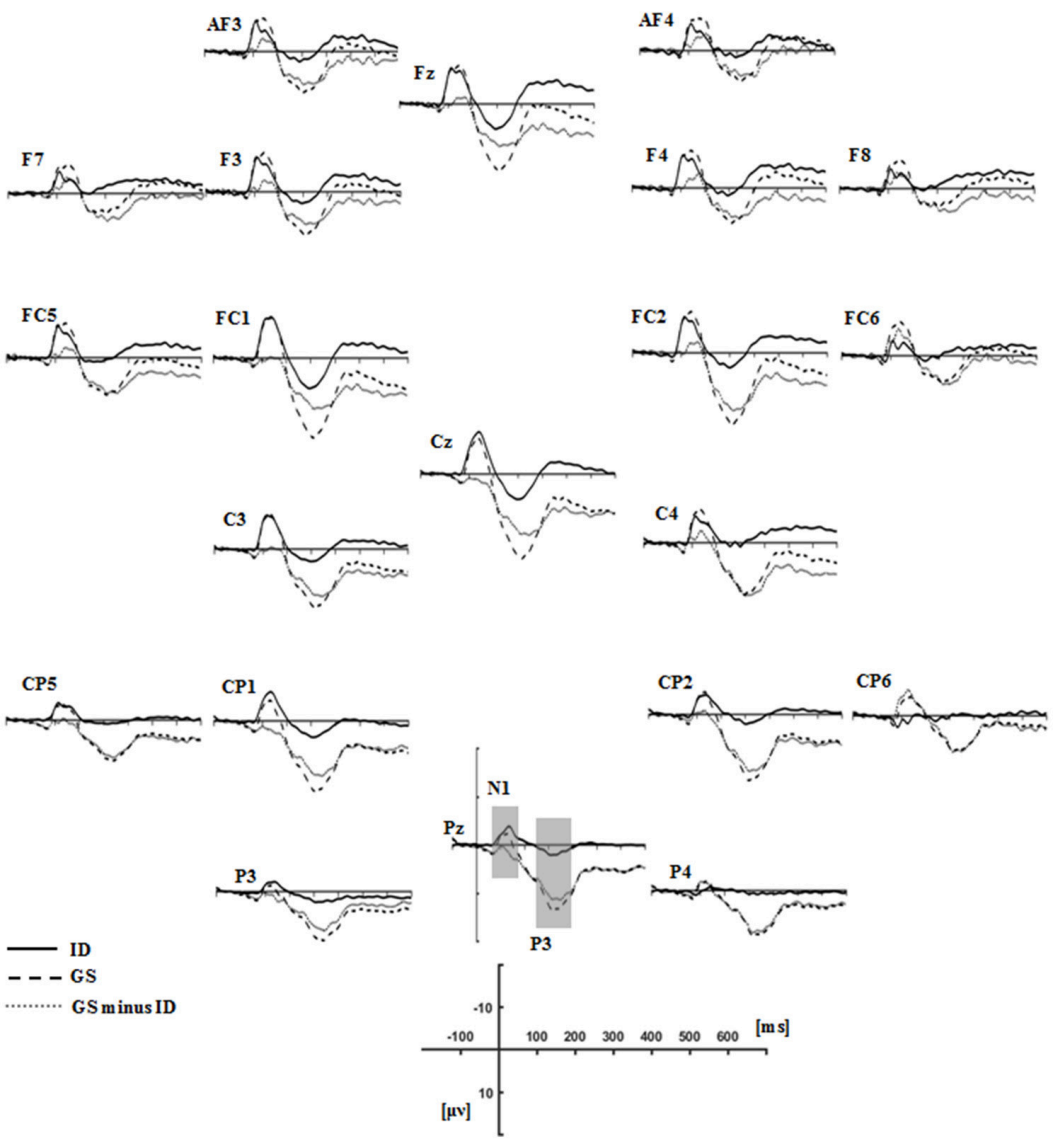
Grand average ERPs time-locked auditory stop-signal stimuli in the successful-stop trials for the groups of insomnia disorders (ID, solid lines), good sleepers (GS, dash lines), and their difference waves (GS minus ID, gray dot lines). N1 (80–180 ms) and P3 (230–400 ms) components were labeled at the site where their peak latencies were determined.

**Figure 4:**
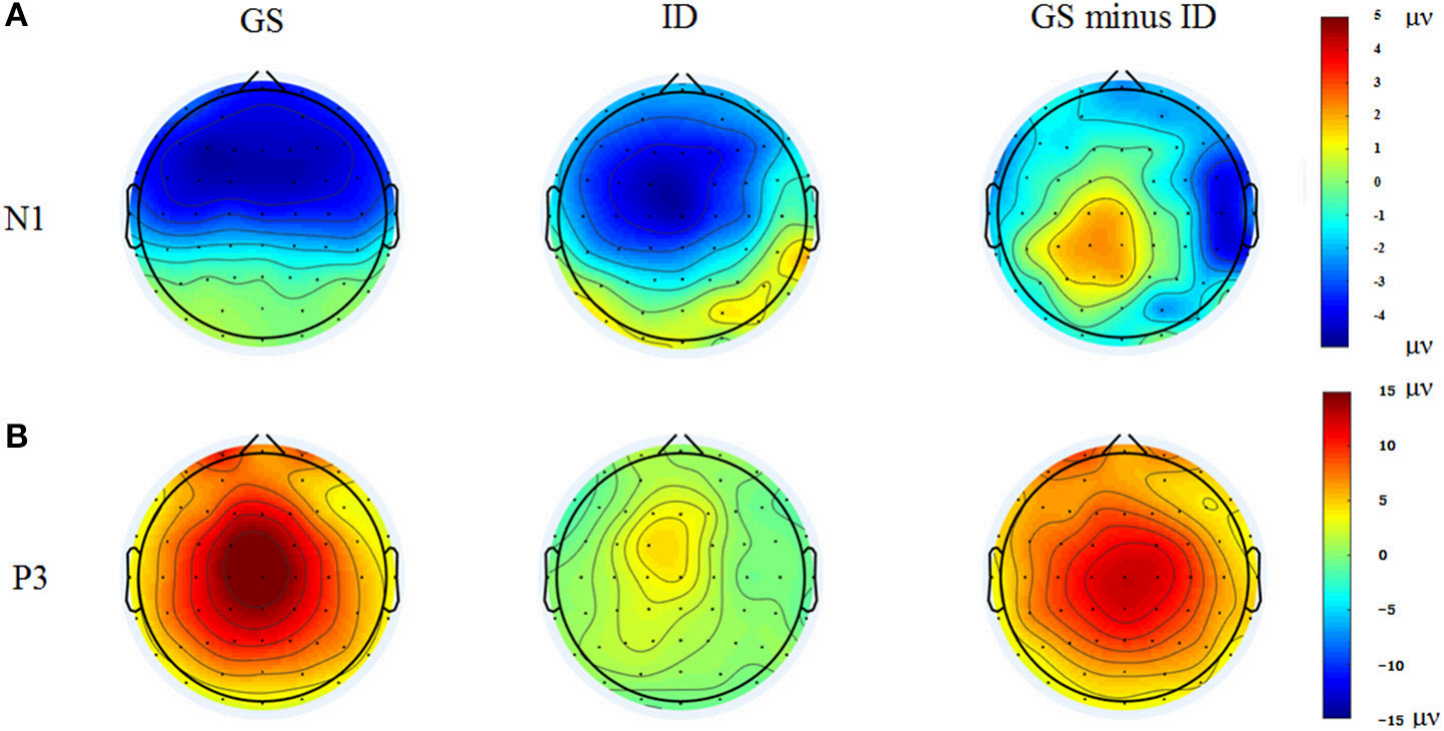
Topographic maps of the N1 (80–180 ms, **A**) and P3 (230–400 ms, **B**) components time-locked to the auditory stop-signal in the successful stop trials. Left: Topographic maps of each ERP component for the group of good sleepers (GS). Middle: Topographic maps of each ERP component for the group of insomnia disorders (ID). Right: Topographic maps of each ERP component for the GS minus ID.

Figure 3 presented each group's grand average ERPs locked to auditory stop-signal in the successful stop trials, and their ERP difference waves were plotted with gray dot lines. N1 (80–180 ms) and P3 (230–400 ms) were the components that we concerned. As for N1 amplitude, results given by repeated measure ANOVA on mean amplitude presented no significant effect. Although there was no significant group or group × ROIs interaction effect when considering N1 latency, significant main ROIs effect was found [*F*_(3.26, 68.44)_ = 4.227, *p* = 0.007, η^2^ = 0.168] (Figure 4A).

We found significant main group effect [*F*_(1, 21)_ = 14.04, *p* < 0.001, η^2^ = 0.401], and marginally significant group × ROIs interaction effect [*F*_(1.80, 37.85)_ = 5.123, *p* = 0.013, η^2^ = 0.196] in P3 amplitude. During the *post-hoc* comparisons, the P3 amplitude in patients with insomnia was significantly smaller than GSs in all ROIs except for the frontal area. The P3 amplitude reached its maximum in the midline and centro-parietal area in GSs, but individuals with ID only exhibited midline dominance (Figure 4B).

### Exploratory analyses of response-locked and stimulus-related ERPs

**Figure 5:**
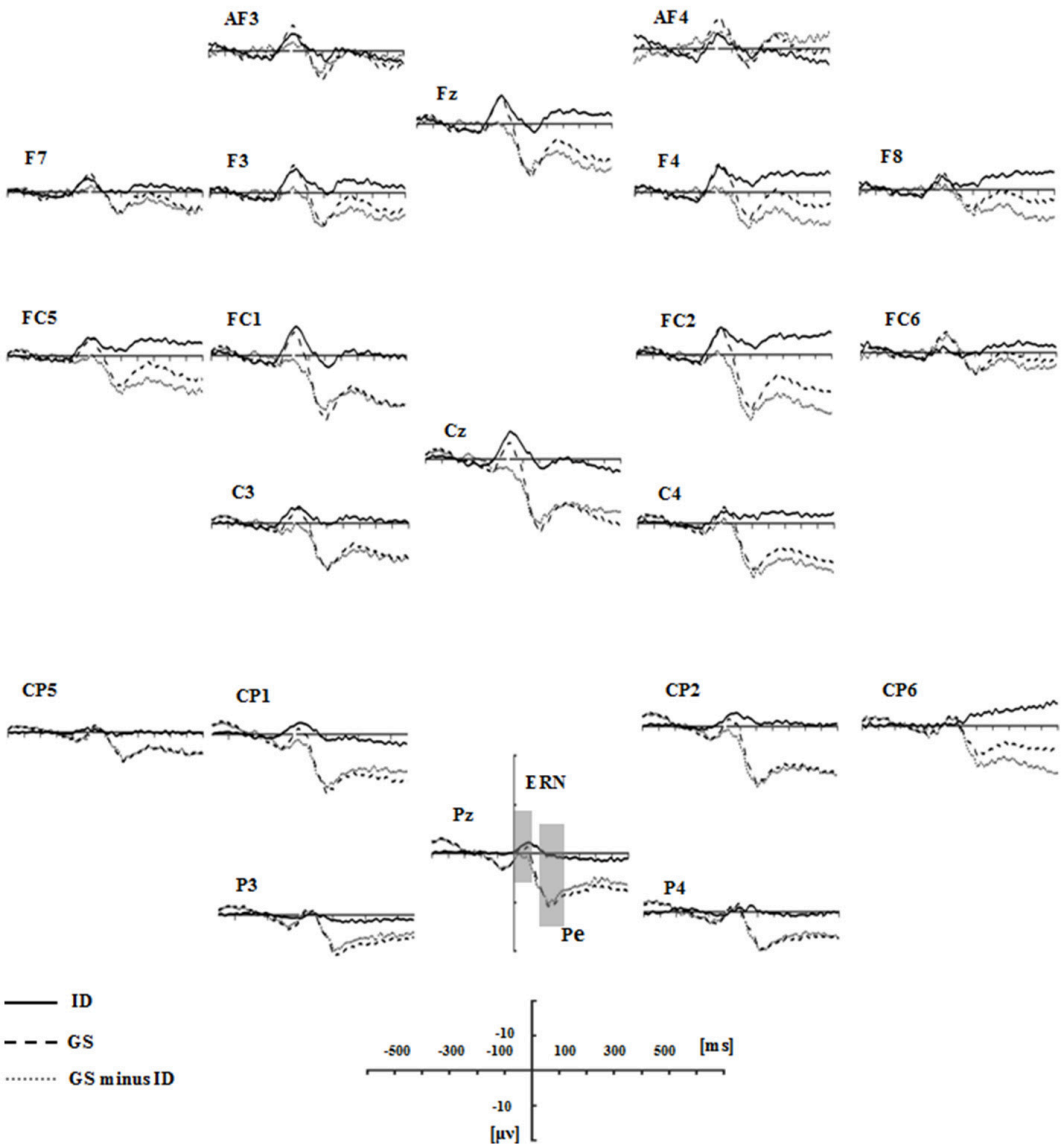
Grand average response-locked ERP waveforms in the failed stop trials for the groups of ID (solid lines), GS (dash lines), and their difference waves (gray dot lines). ERN (0-100 ms) and Pe (150–300 ms) components were labeled at the site where their peak latencies were determined.

**Figure 6:**
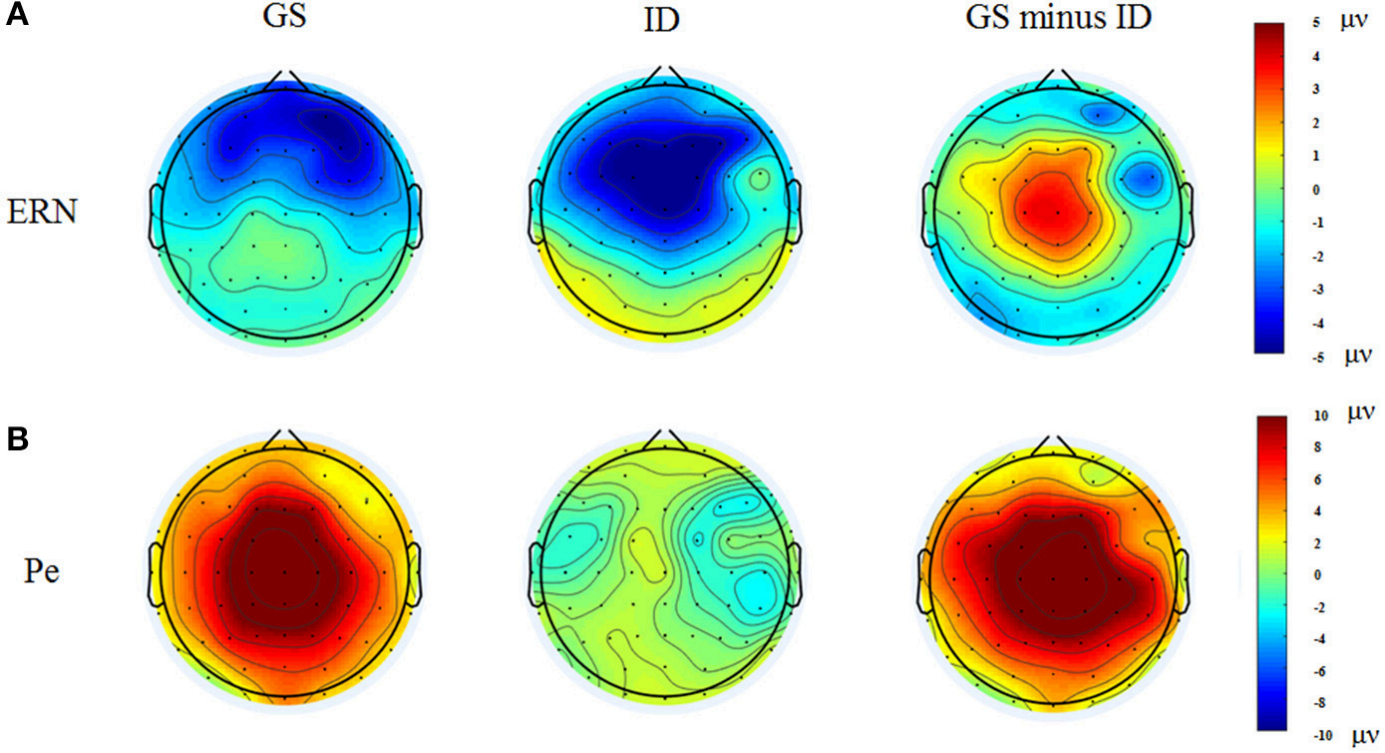
Topographic maps of the ERN (0–100 ms, **A**) and Pe (150–300 ms, **B**) components time-locked to button press in the failed stop trials. Left: Topographic maps of each ERP component for the group of good sleepers (GS). Middle: Topographic maps of each ERP component for the group of insomnia disorders (ID). Right: Topographic maps of each ERP component for the GS minus ID.

Figure 5 presented the grand average ERPs of each group when time-locked to button press in the failed-stop trials. Scalp distribution maps of every component were shown in Figure 6. We found a negative potential just on or after the onset of an overt response, followed by a positive potential. They were known as the ERN-Pe complex. It showed no significant main group effect, both in amplitude and latency of ERN. However, we found marginally significant main group effect [*F*_(1, 21)_ = 7.365, *p* = 0.013, η^2^ = 0.26] in Pe amplitude. *Post-hoc* comparisons indicated that Pe amplitude was significantly smaller in IDs than GSs in all ROIs except for the frontal area. In addition, only the group of GSs presented central-midline dominance, while the insomnia group didn't (Figure 6B).

We also investigated the components of N1 (80–180 ms) and P3 (230–400 ms) in go trials to explore the neuro-mechanism underlying the longer reaction time. As a result, significant main group effect was detected in N1 amplitude [*F*_(1, 21)_ = 11.274, *p* = 0.003, η^2^ = 0.349], while we didn't find the similar result in P3 amplitude [*F*_(1, 21)_ = 1.157, *p* = 0.979, η^2^ = 0.000]. Neither main ROIs nor group × ROIs interaction effect was found in N1 or P3 amplitude. During the *post hoc* comparisons, insomnia group presented significantly smaller N1 amplitude than GSs in all ROIs except for the frontal area.

### Correlations between behavioral parameters and P3 component

In our study, the key finding associated with the impairment of motor inhibition in patients with ID was the reduced P3 amplitude in the successful stop trials. To further investigate the factors that influenced P3 alteration, we explored the partial correlations between the participants' P3 mean amplitude of all selected ROIs except for the frontal area and their behavioral parameters, with age and education as the covariates. We found that P3 mean amplitude was negatively correlated with the score of PSQI (*r* = −0.614, *p* = 0.002), BIS (*r* = −0.428, *p* = 0.041), BIS-M (*r* = −0.438, *p* = 0.036), SAS (*r* = −0.58, *p* = 0.004), SDS (*r* = −0.512, *p* = 0.013). These results suggested that the abnormal P3 amplitude was predictable based on participants' subjective sleep quality, impulsive personality characteristic or emotional state. All the correlations passed the statistical correction after the complementary analyses of bootstrapping tests.

## Discussion

To address whether individuals with ID would present response inhibition deficits, we used the auditory SST to assess the participants' response inhibition process based on their task performance, and further investigated the electrophysiological correlate underlying the performance. Both behavioral and ERPs results revealed the impaired motor inhibitory processing in patients with ID.

### Psychometrics and behavioral performance results

The psychometric data revealed that the two groups differed significantly in the subjective sleep measure (PSQI), the objective sleep measure (WASO), and the emotional indicators (SAS and SDS). These results revealed poorer sleep quality and worse emotional states among patients with insomnia. Although we found no obvious between-group difference in the reaction time of the go trials, SSRT was found to be significantly longer in the group of IDs. These findings, obviously, reflected the impaired motor inhibition in patients with ID, but their motor response was relatively intact. Nonetheless, the relationship between response inhibition and insomnia disorder in prior studies seems to lack consistency. A study used the auditory stop-signal paradigm to corroborate the response inhibition deficits among insomniacs and also found the same kind of longer SSRT as reported in our study (30). Another study, however, came to a contrary conclusion. The study investigated the response inhibition both in patients with ID and OSAS and it claimed that only patients with OSAS showed a longer SSRT, rather than the insomniacs (29). The inconsistency may correlate to the clinical heterogeneity or the inconsistent estimation of SSRT, which can be calculated by subtracting SSD from the finishing time of the stop process or the mean reaction time of the go trials (21, 52). Moreover, functional neuroimaging evidence supported the fronto-basal-ganglia circuit in the implementation of response inhibition (17, 31, 53). In this circuit, via interactions with the subcortical areas, the frontal lobe was proposed to top-down regulate the motor cortex. Therefore, prefrontal atrophy and hypoactivation in insomniacs seem to be the neural correlate of the damaged motor inhibition (15, 16, 36, 37).

### ERP results

Our key finding was the reduced successful stop P3 amplitude in patients with ID. It was suggested that the central-maximum P3 reflected the inhibitory processing and the efficiency of inhibitory control (40, 42). Critically, the dipole source analysis in previous studies pointed that the successful stop P3 might present a source in the primary motor cortex (PMC) (42, 54). PMC was found to be the last cortical area of inhibitory processing and its communication with the prefrontal cortex underpinned the inhibition behavior (55). Whereas this communication was disrupted by the abnormal prefrontal cortex and then patients with insomnia failed to suppress the go response because of their impaired response inhibitory circuit. What's more, the negative correlations between P3 amplitude and the global score of BIS, motor impulsiveness subscale of BIS, as well as PSQI provided convincing evidence for the above-mentioned hypotheses. And more importantly, previous research had revealed that P3 amplitude was found to be smaller in the longer reaction time group than the shorter group (40). This was consistent with our findings, in which insomniacs presented longer reaction time and decreased P3 amplitude in the central-midline area.

However, the observed between-group effect in P3 amplitude in our current study might partly be triggered by the inclusion of go trials, which influenced the process of the stop-signal. Future studies should consider other tasks (for example, two-choice oddball paradigm) that could extract the pure electrophysiological correlate associated with response inhibition.

A negative/positive complex known as the ERN-Pe complex in the failed stop trials can be observed after a button press, which would happen if the inhibition processing failed to stop the primary go response (44, 56). Specifically, the ERN had a noticeable association with error detection, while Pe had been interpreted as the affective processing or a compensatory action to an erroneous response (44, 57). Consequently, failing to inhibit a response in the failed stop trials also evoked the error-related brain potential (i.e., the ERN-Pe complex) in our study. It has to be noted that we found no group difference in both ERN amplitude and latency. This result was inconsistent with some previous studies, which focused on the psychiatric disorders such as anxiety with comorbid insomnia (58), major depressive disorder (59), generalized anxiety disorder (60). Why there was no between-group effect in our study? One possible explanation was that abnormal error-processing had an intimate relationship with trait-related disorders (61). Insomnia-related complaints, however, were found to significantly correlate to state anxiety (62). When it comes to the second component of the ERN-Pe complex, we found that Pe amplitude was significantly decreased in the group of IDs than GSs. The putative neural generator of Pe was located in the rostral anterior cingulate cortex (rACC) (63). The fMRI studies suggested that ACC mainly served as a generic detector for response conflict and action monitoring (64, 65). Consequently, the reduced Pe amplitude among insomniacs reflected their disordered affective processing to the erroneous response. And its reduction might associate with the structural abnormality of rACC, as reported by a previous neuroimaging study (66).

Finally, compare to GSs, we found longer reaction time in the group of IDs, though the between-group difference didn't reach a significant level. However, during the exploratory ERP analyses in the go trials, we found reduced N1 amplitude in insomniacs. As an exogenous component, N1 typically peaked around 100 ms and reflected the early sensory activation and the selective attention to the go stimulus (41). Decreased N1 amplitude suggested the deficient stimulus perception or selective attention among insomniacs. Additionally, a previous ERP study investigating the effect of mental fatigue on attention had focused on a phenomenon known as N1 decrement: the longer the time participants spent on performing tasks, the smaller the negativity was in the N1 amplitude (67). Mental fatigue resulted in a reduction in the goal-directed attention, leaving subjects performing in a more stimulus-driven fashion. Insomniacs presented well-documented fatigue and mood disorders, which had been demonstrated to associate with the integration of prefrontal cortex (68–71). In fact, the prefrontal abnormality in patients with insomnia had been repeatedly confirmed by many studies (15, 16, 36, 37). Consequently, the abnormal prefrontal cortex and insomnia-related mental fatigue might be the reasons why insomniacs presented decreased N1 amplitude in the go trials.

Obviously, our present study has some limitations. Firstly, there were marginally significant between-group differences in age and education level, which might be the possible confounders although they were used as covariates in all statistical analyses. Secondly, owing to the small sample size, statistical power was relatively weak and any inference based on this study should be cautious. Thirdly, emotional disorders like anxiety and depression might be the factors that influence insomniacs' response inhibition process, though we enrolled the patients with mild depression and anxiety. Finally, it was still in controversy about whether the response-locked Pe was completely distinct from the classical P3 in functional significance (43), so any interpretations concerning this component should be taken with caution.

## Conclusions

In conclusion, through using the auditory stop-signal paradigm coupled with ERPs, we carried out this study to examine whether individuals with insomnia presented response inhibition deficits and further investigated the electrophysiological correlate underlying these deficits. We made efforts to verify whether insomniacs presented significantly longer SSRT compared to GSs. The results, in line with prior studies, substantiated the hypothesis that insomnia patients presented deficient motor inhibition, but their motor response was relatively intact. Specifically, the group of IDs presented reduced P3 amplitude in the successful stop trials, indicating the decreased efficiency of response inhibitory processing among insomniacs. Although there was no between-group difference of reaction time in the go trials, the reduced N1 amplitude might still reflect the damaged sensory activation or selective attention to primary stimulus among insomniacs. Finally, with regard to response-locked ERPs, patients with insomnia presented reduced Pe amplitude, suggesting the abnormal affective processing when they made an erroneous response. Taken together, the above-mentioned findings showed that patients with ID usually presented response inhibition deficits and the electrophysiological correlate underlying these deficits typically focus on the successful stop P3 component.

## Author contributions

WZ, DG, and XL designed and performed the experiments. WZ, XL, FY, YW, and DM analyzed the data. WZ and XC wrote the manuscript.

### Conflict of interest statement

The authors declare that the research was conducted in the absence of any commercial or financial relationships that could be construed as a potential conflict of interest.
